# An explorative cross-sectional study on the association between immunological biomarkers in patients with chronic pain

**DOI:** 10.1016/j.bbih.2026.101339

**Published:** 2026-08-29

**Authors:** Jenny Hadrévi, Johanna Philipson, Britt-Marie Stålnacke, Nils Berginström, Monika Löfgren, Anna Holmqvist, Marika C. Möller, Bijar Ghafouri

**Affiliations:** a Department of Public Health and Clinical Medicine, Family Medicine, Umeå University, Sweden; b Department of Clinical Sciences, Neuroscience, Umeå University, Sweden; c Department of Community Medicine and Rehabilitation, Rehabilitation Medicine, Umeå University, Sweden; d Department of Psychology, Umeå University, Sweden; e Department of Clinical Sciences, Karolinska Institutet, Danderyd Hospital, Sweden; f Department of Rehabilitation Medicine Stockholm, Danderyd University Hospital, Sweden; g Department of Health, Medicine and Caring Sciences, Linköping University, Sweden

**Keywords:** Chronic pain, Fatigue, Immunological biomarkers

## Abstract

**Background:**

Studies indicate an association between pain perception and inflammatory response. However, the expression of inflammatory cytokines in the regulation of the interaction between pain and other coexisting symptoms related to pain, such as fatigue, remains largely unknown.

**Aim:**

This study utilizes a comprehensive screening method for inflammatory biomarkers to objectively study their relation to fatigue and other patient reported outcomes, such as depression and sleep disturbances, in a cohort entailing patients with chronic pain and controls reporting no pain.

**Material and methods:**

36 patients with chronic pain and 36 healthy pain free controls were included. To investigate possible differences between patients and controls, the QUICKPLEX SQ 120 with a panel of 71 pro- and anti-inflammatory proteins was used. Patient reported data covering aspects of fatigue, fatigability, mood and sleep was also administered.

**Results:**

No apparent difference in abundance of inflammatory substances was found between patients and controls. Likewise, no association was between levels of inflammatory biomarkers and performance on objectively measured cognitive fatigability. However, an explorative multivariate PCA analysis with immunological response as the determinant revealed five specific clusters, two clusters of controls and three patient clusters. One patient cluster had an overall lower concentration level of inflammatory substances compared to the other clusters. This cluster reported higher levels of general fatigue (trait fatigue) and state fatigue as well as higher proportions of pain localizations, perceived helplessness, insomnia, depressive symptoms, and anxiety.

**Conclusion:**

This study underscores the complexity of pain perception and its interplay with inflammatory responses, as well as the individual experience of fatigue and other co-occurring symptoms. We argue that patients with chronic pain do not constitute a homogeneous group, and that simple patient–control comparisons may be insufficient to elucidate the underlying inflammatory processes involved. Our findings suggest that chronic pain patients who report higher levels of fatigue, sleep disturbances, and depressive symptoms might exhibit inflammatory response proteins associated with tissue-repair mechanisms. We speculate that this pattern could indicate a response driven more by central pain mechanisms.

## Highlights

• Chronic pain patients showed no overall differences in inflammatory biomarkers compared with pain-free controls.
• Fatigue, sleep disturbances, anxiety, and depression were linked to distinct inflammatory profiles.
• Multivariate biomarker profiling revealed biologically distinct subgroups among patients with chronic pain.
• A patient cluster with lower inflammatory protein levels reported greater fatigue and symptom burden.
• Findings suggest that chronic pain patients may not exhibit uniform inflammatory profiles, emphasizing the potential role of neuroplastic mechanisms.

## 1 Introduction

One of the most frequently presenting conditions in healthcare is pain (Mäntyselkä et al., 2003; Finley et al., 2018). When present for more than 3 months, the condition is classified as chronic pain (Nicholas et al., 2019). Chronic pain has been theoretically explained to be attenuated and maintained by affecting the descending and ascending pathways in the central nervous system (CNS), creating a neuronal imbalance facilitating pain transition and reducing pain inhibition (Ossipov et al., 2014; Yam et al., 2018). This may lead to plastic changes in the CNS, resulting in increased sensitivity to pain and increased risk for depression, anxiety and fatigue. (Flodin et al., 2015; Marchand, 2008; Bartley et al., 2018).

Chronic low-grade inflammation has been suggested to play a role in the maintenance of chronic pain and elevated levels of inflammatory biomarkers have been reported in patients with neuropathic pain and generalized chronic pain (Gerdle et al., 2020; Jönsson et al., 2021; Olausson et al., 2015a; Hysing et al., 2019). Significant correlations between proteins related to inflammatory response and psychological distress in patients with fibromyalgia have also been reported (Wåhlén et al., 2020). Unresolved inflammation and/or repeated long-term stimulation of the immune system can lead to systemic chronic inflammation. In contrast to an acute inflammatory reaction, systemic chronic inflammation is characterized by a long-lasting, low-grade profile usually defined as a low-grade state of inflammatory factors that originate from several physiological mechanisms. Systemic inflammation has been identified in most pain states (Åström Reitan et al., 2024) and has collateral effects on multiple systems and organs, even though several established biomarkers for acute inflammation are not detectable, or barely detectable in this condition (Furman et al., 2019; Sturmberg et al., 2017). This kind of low-grade profile of inflammatory factors has also been demonstrated in chronic pain (Rosenström et al., 2024). Differences in levels of inflammatory biomarkers have been reported when comparing patients with healthy controls at the group level, but the levels are still within the healthy range at the individual level. For example, CRP levels in patients with chronic pain remain within the reference interval. These findings need corroboration, which would be difficult to reach efficiently using only hypothesis-driven approaches with only a few markers (Karshikoff, 2024).

Mental fatigue is highly prevalent in chronic pain and often described as a state of constant exhaustion and depression (Wolfe et al., 1996; Mease et al., 2007; Fishbain et al., 2003). Since fatigue often interferes with other coexisting conditions, also common in patients with chronic pain, such as depression, anxiety, and sleep disturbances, it can be difficult to determine whether the reported fatigue is primarily related to pain, or secondary to other coexisting conditions (Bartley et al., 2018; Mease et al., 2007; Snekkevik et al., 2014; Arnold, 2008; Akerstedt et al., 2004; Hiestand et al., 2023).

Fatigue may arise both from non-restorative sleep and is a central symptom of depression (Crouse et al., 2021) and further exacerbated by persistent pain that impairs restorative rest (Haack et al., 2020). Disturbed sleep is furthermore a core feature of depression and prospectively increases risk for its onset (Baglioni et al., 2011), as depression disrupts sleep architecture, circadian stability, and cognitive–emotional arousal, reinforcing sleep–mood dysregulation (Franzen et al., 2008; Xing et al., 2025). Neurobiological evidence suggests shared pathways, including dysregulation of the hypothalamic–pituitary–adrenal (HPA) axis, altered monoaminergic neurotransmission, and circadian rhythm disruption (Dollish et al., 2024). Sleep disturbance has also been suggested to be linked to elevated inflammatory markers such as IL-6 and CRP, which contribute to depression and sickness-related fatigue (Ghilotti et al., 2021; Irwin et al., 2016). Proinflammatory cytokines can alter neurotransmitter metabolism and microglial activation, driving symptoms such as anhedonia, hyperalgesia, and fatigue (Dantzer et al., 2008). Thus, previous research substantiates a multilevel, reciprocal model in which fatigue, sleep disturbances, depression, pain, and inflammation dynamically reinforce one another over time.

Mental fatigue is frequently measured by self-report questionnaires, often asking for ratings of fatigue over a period of time, thus representing the person's predisposition for fatigue. This is commonly referred to as *trait fatigue* (Wylie et al., 2017). The Multidimensional Fatigue Inventory (MFI-20) is a widely used rating scale to measure trait fatigue and consists of five subscales probing for different aspects of fatigue, which will be elaborated further in the methods section. Trait fatigue is, however, often highly correlated to depression (Holmqvist et al., 2024). Another aspect of mental fatigue is *state* fatigue, which refers to a more temporary and fluctuating sensation of exhaustion or tiredness experienced in a specific moment or situation. State fatigue can for instance occur while performing a cognitively demanding task (Wylie et al., 2017). To subjectively quantify state fatigue and energy levels at a specific time point, the Visual analog scale (VAS) can be used and give an indication of state fatigue (Wylie et al., 2017) before, and after performing a cognitively demanding task. Furthermore, performance decline on such tasks is generally referred to as cognitive fatigability (CF). At a functional level this can be operationalized as an inability to maintain performance during a continued complex information processing task (Bryant et al., 2004; Linnhoff et al., 2019; Schwid et al., 2003; Walker et al., 2019).

Studies on patients with different neurological conditions and brain injury have shown that CF can be induced during cognitively demanding tasks. CF has also been suggested to be a free-standing phenomenon and possibly more evident in cognitively preserved individuals, rather than in those who are cognitively impaired. It has been found not to be influenced by the presence of depression (Tommasin et al., 2020). Recent fMRI results indicate that CF is present in patients with chronic pain and is associated with increased activation in brain regions related to motor and cognitive control (Holmqvist et al., 2025). However, it remains to be determined if the presence of cognitive impairment may mask the occurrence of CF, or if CF precedes cognitive impairment.

Sleep disturbances are common in chronic pain (Todd et al., 2022), and a major contributor to fatigue (Lichstein et al., 1997) and mood disturbances (Liu et al., 2025). Sleep deprivation affects the ability of the cerebral cortex to perceive the pain threshold and increases pain sensitivity (Haack et al., 2020). Regulation of sleep, wake and nociception mechanistically share common neuroanatomic and molecular substrates (Chen et al., 2024). A possible link between systemic inflammation and sleep deprivation has been presented (Mullington et al., 2010; Faraut et al., 2012). However, the contribution of neurotransmitters, opioid systems and inflammatory cytokines in the regulation of the interaction between pain, mental fatigue, and sleep remains largely unknown (Kourbanova et al., 2022), though pain in itself often induce both fatigue and sleep disturbances (Smith et al., 2004).

Animal studies (Dai et al., 2022) have suggested that chronic sleep deprivation may aggravate pain behavior in rats with chronic pain through the TLR4/NLRP3/IL-1b signaling pathway. This may in turn mediate microglial M1 polarization, increase CNS inflammation, and promote neuronal apoptosis. It has therefore been suggested that microglial activation caused by sleep deprivation may play an important role in the aggravation of chronic pain (Dai et al., 2022).

Another recent study indicated that chronic sleep deprivation aggravated lipopolysaccharide-induced anxiety, depression, and cognitive impairment. These adverse outcomes may be the result of changes in pro-inflammatory cytokines and synaptic plasticity associated proteins induced by chronic sleep deprivation (Zhang et al., 2024).

In recent years, some studies have expanded the screening for inflammatory biomarkers using multiplex panels for inflammation-related blood proteins (i.e. cytokines, chemokines and growth factors). Elevated levels of inflammatory biomarkers have been reported in patients with neuropathic pain and generalized chronic pain (Hysing et al., 2019; Gerdle et al., 2017, 2019) as well as ongoing inflammatory processes in pain patients (Åström Reitan et al., 2024). Other studies have reported differences in abundance of inflammatory biomarkers in patients with chronic pain (Hysing et al., 2019; Bäckryd et al., 2017) compared to healthy controls. Thus, recent studies indicate an association between pain perception and inflammatory response. However, the expression of inflammatory cytokines in the regulation of the interaction between pain and other coexisting symptoms related to pain, such as fatigue and sleep disturbances, remains largely unknown.

This gap in knowledge limits our understanding as to why certain individuals develop more pronounced and persistent symptoms, which is why this study took an explorative approach and did not focus on specific markers. Thus, by examining inflammatory biomarkers within a framework that accounts for the interactions between pain, fatigue, and co-occurring symptoms, we aim to advance insight into the underpinnings of long-term pain and thereby support the development of more targeted therapeutic strategies.

## 2 Aim

In this explorative cross-sectional study, we utilize a comprehensive screening method for inflammatory biomarkers to objectively study their relation to fatigue and fatigue related patient reported outcomes in a cohort entailing patients with chronic pain and controls reporting no pain.

## 3 Material and methods

The present cross-sectional study is a sub-study of a larger clinical trial (ClinicalTrials.gov NCT05452915) (Möller et al., 2023). The study was approved by the Swedish Ethical Review Authority (2018/424-31; 2018/1235–32; 2018/2395–32; 2019-06148; 2022-02838-02).

### 3.1 Participants

#### 3.1.1 Inclusion criteria

Based on the definition of chronic pain established by the International Association for the Study of Pain (IASP) (Treede et al., 2015), participants were required to have been presented with persistent or recurrent pain for more than three months. They were to be between 18 and 45 years of age and referred either to the Department of Rehabilitation Medicine at Danderyd University Hospital or to the Department of Pain Rehabilitation, Pain Center, Umeå University Hospital. An upper age limit of 45 years was set to minimize the influence of age-related comorbidities on chronic pain.

#### 3.1.2 Exclusion criteria

Individuals were excluded if they had a traumatic brain injury (including concussion) or any other neurological disorder (including ME/CFS). Additional exclusion criteria included extensive psychiatric problems or substance abuse, a history of congenital or acquired brain injury, not living in their own home or needing assistance in daily life, insufficient knowledge of the Swedish language, and the presence of a progressive disease. The use of drugs with a strong sedative effect and pregnancy also led to exclusion. Because some participants underwent an fMRI investigation as part of another sub study (patients: n = 24 and controls n = 22), further exclusion criteria applied for those individuals: metal implants or metal chips in the body, claustrophobia, and left-handedness. For the control group, chronic or current pain constituted additional exclusion criteria.

### 3.2 Procedure

All patients referred to the Pain Center at Umeå University Hospital or the Department of Rehabilitation Medicine at Danderyd University Hospital Sweden, from September 2020 to September 2021 were asked to participate in the larger cross-sectional study (Möller et al., 2023). In the present study, 36 consecutive patients underwent blood sampling for immunological markers. The patients were consecutively included until we reached a final and sufficient sample size. Based on previous biomarker studies (Jönsson et al., 2021; Ghafouri et al., 2022) the sample size in this study should be sufficient to detect a difference between groups. To ensure that all samples could be processed simultaneously and thereby minimize potential batch effects associated with running multiple panels at different time points, we included 36 patients and 36 controls. This sample size therefore also allowed all samples to be analyzed within a single panel run, reducing methodological variability. Measurements probing cognitive fatigability and self-rated state fatigue/fatigability were also collected, as well as self-reported data on symptoms of depression, anxiety, sleep and physical activity. All patients were diagnosed with chronic pain for more than three months. The five most frequent diagnosis were: M35.7 Hypermobility Syndrome (n = 24), M79.7 Fibromyalgia (n = 16), Q79.6 Ehlers-Danlos syndrome (n = 19), R52.2C Other chronic pain (n = 15), and R52.9 Pain, unspecified (n = 19) which account for 46.5% of the patient group. The following conditions/diagnoses were present: Chronic neck–shoulder pain (cervicalgia, trapezius myalgia), chronic low back pain without sciatica, generalized pain (according to the American college of Rheumatology (ACR) 2016 criteria), Fibromyalgia (according to the ACR 2016 criteria), Neuropathic pain including sciatica, Chronic whiplash disorders, Ehlers-Danlos syndrome or hypermobility syndrome, Complex regional pain syndrome, Pain post-COVID infection, Chronic pain, not elsewhere classified (ICD R52.1, R52.2 and R52.9). Although the included diagnoses may vary, chronic pain was treated according to the International Association for the Study of Pain (IASP) which recognizes chronic pain lasting longer than 3 months not just a symptom, but as a disease in its own right (Treede et al., 2019).

**Fig. 1:**
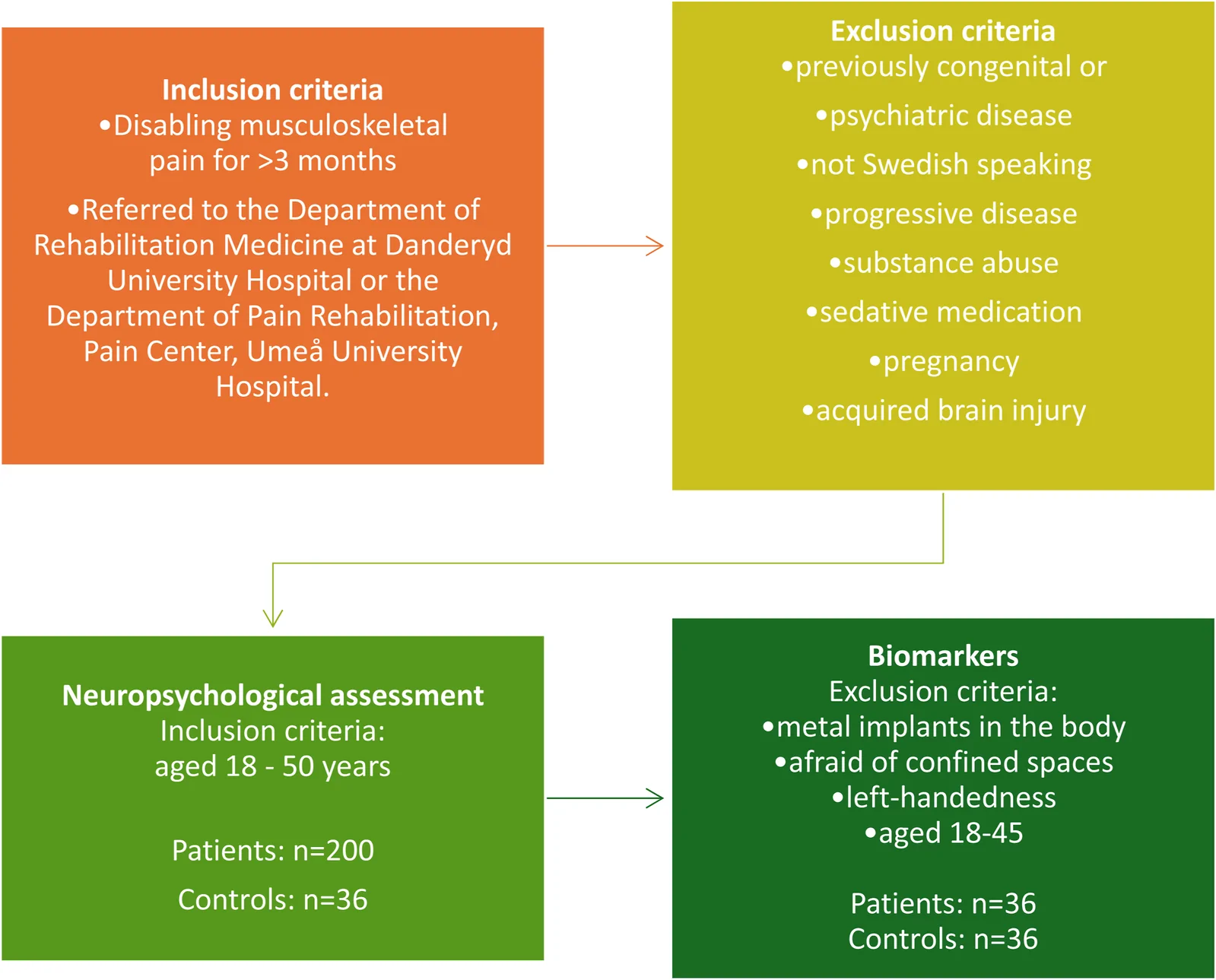
Study flowchart.

Of the subjects eligible for blood sampling, 21 were excluded due to fulfilling one or several exclusion criteria, mainly concussion, not speaking Swedish or medical treatments interfering with cognitive function. Two participants were excluded due to technical errors during blood sampling. See Fig. 1 for detailed flow-chart.

A convenience sample of 36 healthy controls was recruited through advertising among hospital staff. The controls were matched on a group level regarding age, and level of education. All participants provided written informed consent to participate in the study.

### 3.3 Blood sampling and biochemical analysis

To minimize systemic variation, fasting venous blood samples (200 μl) were collected at 7.30 a.m., in P100 tubes from BD diagnostic, containing a protease inhibitor cocktail that prevents protein cleavage/degradation from each subject. All subjects were instructed not to engage in any physical exercise prior to the blood sampling. The blood samples were centrifuged to remove red blood cells. The plasma fraction was transferred to a new tube, portioned and stored at −86°C until analysis at the PAINOMICS lab in Linköping, Sweden. Plasma samples were analyzed using the antibody-based method multiplex technology (Meso Scale Diagnostics, Rockville, MD). The commercially available panel of 71 pro- and anti-inflammatory proteins (cytokines, chemokines, and growth factors) (U-PLEX, Meso Scale Discovery, Maryland, USA) was used (Supplementary Table 1). This analytical method provides the absolute concentration. Therefore, there is no need for additional normalization of data. On the day of analysis plasma samples were thawed and analyzed according to the manufacturer protocol. The light intensity of all the different proteins examined was converted into concentrations (pg/ml) using an 8-point standard curve with known concentrations of each protein. Data was collected and analyzed using MESO QUICKPLEX SQ 120 instrument equipped with DISCOVERY WORKBENCH data analysis software.

### 3.4 Measurements

The Swedish Quality Register for Pain Rehabilitation (SQRP) (http://www.ucr.uu.se/nrs/) is a national quality register for adult patients with complex chronic pain conditions developed in 1998 to ensure quality of pain care services and to develop evidence-based methods in pain rehabilitation in Sweden. The registry has been implemented in over 40 specialty clinics in Sweden and is used both for quality control in health-care management and for clinical research. Using validated questionnaires, the data collected in SQRP monitors patient-related outcome measurements regarding (for example) insomnia (Insomnia Severity Index; ISI), depression and anxiety (Hospital Anxiety and Depression Scale; HADS), perceived quality of life (EuroQol-5 dimensions questionnaire), screening on physical activity, pain intensity (Numeric Pain Rating Scale; NPRS). See below for more detailed description regarding each questionnaire.

*Hospital Anxiety and Depression Scale* (HADS) (Snaith et al., 1986) is a self-assessment scale found to be reliable for detecting states of depression and anxiety in medical outpatients. The subscales (anxiety and depression) are valid measures of severity of the respective emotional disorder.

*Insomnia Severity Index* (ISI) (Bastien et al., 2001) is designed as a brief screening tool for insomnia. The ISI is a seven-item questionnaire where respondents rate the nature and symptoms of their sleep problems using a Likert-type scale.

*Screening on physical activity* by the National Board of Health and Welfare (Kallings et al., 2014) comprising of three questions asking how many minutes during the last week they have performed exercise that makes them breathless, how many minutes they have been otherwise physically active besides exercise, (i.e. housework or gardening), and how many hours they usually sit during a day.

The *Multidimensional Fatigue Inventory-20* (MFI-20) (Manoli et al., 2020) consists of five subscales probing for different aspects of general/trait fatigue: General Fatigue (GF), Physical Fatigue (PF), Reduced Motivation (RM), Reduced Activity (RA) and Mental Fatigue (MF).

*Multidimensional Pain Inventory* (MPI) (Kerns et al., 1985) was used to probe for impact of pain in everyday functioning.

*EuroQol-5 dimensions* questionnaire (EQ-5D) (Group, 1990) which is a descriptive system that comprises five dimensions: mobility, self-care, usual activities, pain/discomfort and anxiety/depression. Each dimension has 5 levels: no problems, slight problems, moderate problems, severe problems and extreme problems.

*Number of pain localizations:* According to the International Association for the Study of Pain (IASP) pain localizations are registered using 36 predefined anatomical areas with pain on the left side of the body (n = 18) and on the right side of the body (n = 18).

*VAS fatigue:* To measure state fatigue, VAS measurement, although not specifically validated for use in patients with pain, has been recommended by Wylie et al. (2017). The anchors for the VAS scale are “No fatigue at all → worst possible fatigue”. Self-rated state fatigue (VAS-f) was conducted before and after neuropsychological assessment, as well as measurement of self-rated state fatiguability. Subjective state fatigability (VAS-f d-value) was obtained by subtracting the VAS-f value obtained before assessment from the value obtained after assessment.

As medications can influence immune function, we also evaluated the use of medications based on self-reports, we also enquired about possible trauma. Background information was gathered from medical records.

### 3.5 Test of cognitive fatigability

Wechsler Adult Intelligence Scale-III (WAIS-III) (Hartman, 2009): Digit-Symbol, Coding, measuring psychomotor speed and incidental memory. Higher values indicate better results. The Coding subtest from WAIS-III was used instead of WAIS-IV as the former has been validated for measuring cognitive fatigability (CF). CF was measured by comparing the performance during the first 30 s and the last 30 s of the 120 s Digit Symbol Coding test (Wechsler, 2003). CF was measured both as a dichotomized and as a continuous variable. The dichotomized variable was defined as a non-ascending score (<0) when the produced numbers during the last 30 s of the task were subtracted from the number produced during the first 30 s. The continuous variable was reported as the percentage of produced numbers during the first 30 s subtracted from the percentage of completed (Wechsler, 1981).

*Pain level*, experienced during the neuropsychological examination was rated (0–10) at the end of the session.

### 3.6 Statistical analysis

For inflammatory biomarker data, multivariate data analysis (MVDA) was performed using SIMCA-P+ version 18.0 (Sartorius Stedim Biotech, Umeå, Sweden) according to Wheelock & Wheelock (Wheelock et al., 2013) and as described previously (Gerdle et al., 2024; Olausson et al., 2015b). As mentioned in the published study protocol (Möller et al., 2023), MVDA works well not only for “long and lean” data tables, but also for “short and wide” (i.e. for situations when number of participants are much less than number of variables). MVDA (in contrast to classical methods of statistics) has the advantage of being able to handle many variables and few observations (short and wide data tables) (Shaffer, 2002). Principal Component Analysis (PCA) was applied as a first step to determine the presence of any multivariate outliers and multivariate correlation patterns using Hotelling's T^2^ range (strong outliers) and distance to model in X-space (moderate outliers). Orthogonal Partial Least Square Regressions discriminant analysis (OPLS-DA) was made for determining group differences. Hierarchical cluster analysis (HCA) was performed on the principal component score vectors using the default Ward linkage criterion to identify subgroups, as described previously (Bäckryd et al., 2022). HCA is a complement to PCA that can identify relevant subjects based on a dendrogram where group belonging is defined statistically by SIMCA. When the clusters based on the biomarkers are identified then the clinical data are compared between clusters to determine the clinical relevance of the identified clusters. The validity of each model was estimated using cross validation. Hence, for each model, we report the *p*-value of a cross-validated analysis of variance (CV-ANOVA), R^2^ and Q^2^. A p-value for CV-ANOVA less than 0.05 was considered statistically significant. The R^2^ value describes the goodness of the fit, and the Q^2^ value describes the goodness of prediction, where R^2^ should be greater than Q^2^, and the difference between them should not exceed 0.3. Proteins with a VIP (Variable of Importance) value > 1 and an absolute p(corr) value > 0.3 were considered statistically significant (Shaffer, 2002). The p(corr) is the loading of each variable scaled as a correlation coefficient (ranging from −1 to +1) where the sign indicates the direction of the relationship (negative or positive).

Independent sample t-tests were used to investigate differences in demographic variables between the patient group and controls. To compare levels of fatigue and fatigability, as well as self-reported measures of anxiety, depression, and physical activity between the patient group and pain free controls, independent samples t-tests were used. A one-way ANOVA was conducted to compare possible effects of inflammatory biomarkers on cognition and self-reported outcomes between the in clusters suggested by the HCA-analysis. All calculations were made using IBM SPSS Statistics 29. Statistical significance level was set to p < 0.05.

## 4 Results

### 4.1 Measures of cognitive fatigability and self-reported data

The demographic data for patients and healthy subjects are summarized in Table 1. No significant differences were found between patients and controls regarding years of education or age. BMI was only calculated in the patient group, and three male subjects were included in the control group. None of the participants in the control group reported taking anti-inflammatory or anti-depressant medication. Among the patients, the majority reported usage of anti-inflammatory medication and/or anti-depressants, such as SSRI and TCA.

**Table 1:** Demographic, clinical measurements and patient reported data for all subjects.

	Patient group	Controls	p-values
Gender distribution (% female)	100	91.67	**0,0002**
Years of education (Mean; SD)	13.99(2.15)	14.63(2.02)	0.482
Age (Mean; SD)	33.47(7.25)	32.97(9.18)	0.109
BMI (Mean; SD)	26.31(6.23)	n.a.	n.a.

When comparing self-reported data and measures of fatigue and fatigability, significant differences were found between the patient group and pain free controls regarding self-rated level of state fatigue (VAS-f 1 and 2) before *t*(70) = -6.808,*p* < 0.001 and after *t*(70) = -7.632,*p* < 0.001 the neuropsychological assessment. There was, however, no significant difference between patients and controls in the objective measure of cognitive fatigability *t*(70) = 0.816, *p* = 0.417, nor when comparing level of fatigability before and after the neuropsychological assessment (*t*(70) = 1.211,*p* = 0.230. Patients, however, reported higher levels of trait fatigue on all subscales on the MFI-20 (all *p* < 0.001) with total MFI-20 *t*(70) = -14.837,*p* < 0.001, as well as symptoms anxiety t(67) = -5.172,*p* < 0.001, depression *t*(67) = -11.481, *p* < 0.001, and sleep disturbances *t*(66) = -9.294,*p* < 0.001. Patients also rated their physical activity as lower compared to controls regarding physical exercise *t*(67) = 4.353,*p* < 0.001, everyday physical activity *t*(67) = 2.528,*p* = 0.007, and sedentary behavior *t*(67) = 1.879,*p* = 0.032 (See Table 2).

**Table 2:** The mean(SD) of clinical characteristics, and patient reported data.

Self-reported measures	Controls Mean (SD)	Patient group Mean (SD)	P-value
VAS-f 1	23.98(15.86)	56.10(23.45)	**<0.001**
VAS-f2	33.54(23.87)	69.74(15.49)	**<0.001**
Cognitive Fatigability	−0,58(6.06)	−1.54(3.56)	0.417
Fatigability (VAS1-VAS2)	−9.44(19.75)	−13.44(16.69)	0.230
MFI Total	38,86(10.22)	75.42(10.26)	**<0.001**
MFI General	8.86(3.0)	17.75(2.02)	**<0.001**
MFI Physical	7.67(3.34)	16.39(3.40)	**<0.001**
MFI Mental	9.11(3.19)	15.58(2.55)	**<0.001**
HAD (Anxiety)	4.56(3.28)	9.58(4.71)	**<0.001**
HAD (Depression)	1.22(1.24)	8.39(3.52)	**<0.001**
ISI (Sum initial)	4.28(2.89)	14.56(5.89)	**<0.001**
Physical exercise	4.14(1.55)	2.48(1.60)	**<0.001**
Everyday physical activity	5.56(1.08)	4.76(1.52)	**0.007**
Sedentary behavior	4.43(0.94)	4.0(1.37)	**0.032**
Number of pain localizations	n.a.	18.36(8.41)	
Perceived helplessness (PCS)	n.a	12.27(5.42)	

### 4.2 Differences in inflammatory proteins in patients compared to controls

To investigate the presence of potential outliers, the concentrations of the 71 cytokines/chemokines in plasma were analyzed from both patients and controls using PCA. One outlier (patient) was detected and removed from further analysis. The remaining subjects, both patients and controls, were analyzed using OPLS-DA. No significant OPLS-DA model could be detected, suggesting that the analyzed inflammatory proteins were not able to discriminate between patients and controls.

### 4.3 Cluster analysis based on inflammatory proteins

**Fig. 2:**
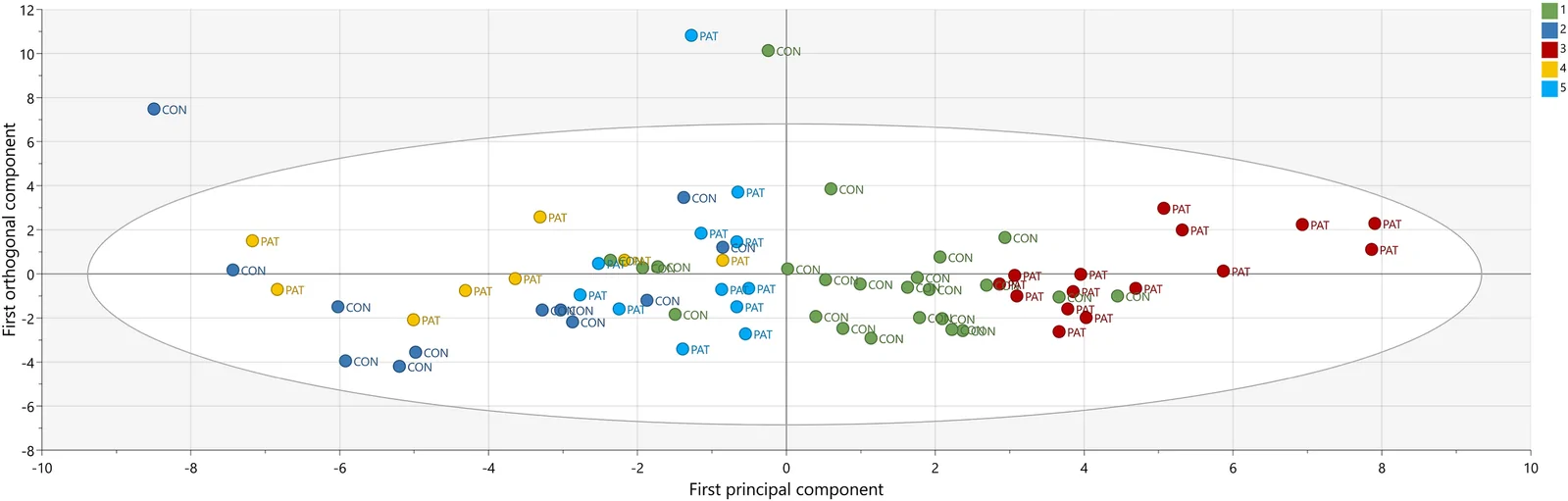
Score plot showing separation between five clusters of subjects (2 controls and 3 patients) using orthogonal partial least square discriminant analysis (OPLS-DA).

Hierarchical cluster analysis (HCA) was performed on the PCA, excluding the outlier. The HCA analysis detected five clusters based on the inflammatory proteins (Fig. 2). Two clusters (1 and 2) included only controls (n = 24 and n = 12), and the remaining three clusters (3,4 and 5) contained patients (n = 14, 7 and 11) (two components, CV-ANOVA p = 0.004, R^2^ = 0.2, Q^2^ = 0.1). See Table 3 for demographic data based on the five clusters.

**Table 3:** Demographic, clinical measurements and patient reported data for all subjects based on the five clusters.

	Cluster 1	Cluster 2	Cluster 3	Cluster 4	Cluster 5	p-values
Patient - Control (% patients)	0	0	100	100	100	n.a.
Gender distribution (% female)	87.5	100	100	100	100	0.195
Years of education (Mean; SD)	14.44(2.33)	15(1.2)	14.37(2.39)	14.1(1.97)	13.63(2.06)	0.684
Age (Mean; SD)	34.21(10)	30.5(7)	32.67(7.1)	31.13(8.92)	36.67(5.61)	0.369
BMI (Mean; SD)	n.a.	n.a.	26.88(7.2)	26.4(6.69)	25.47(4.96)	0.883

The two clusters of controls and the 3 clusters of patients were analyzed separately using OPLS-DA to find significant proteins that discriminate against the clusters. Significant OPLS-DA models for both controls (Two components, CV-ANOVA p = 0.04, R^2^ = 0.7, Q^2^ = 0.3) and patients (Two components, CV-ANOVA p = 3.2x10^−05^, R^2^ = 0.5, Q^2^ = 0.3) were detected (Fig. 3a, Fig. 3ba and b). Cluster 1 and 2 differed in 25 proteins with VIP> 1.0 and absolute p(corr) > 4.0 (Supplementary Table 1). All 25 proteins were significantly upregulated in cluster 2 compared to cluster 1. The 3 male controls were all represented in cluster 1. To correct for possible gender differences, a new OPLS-DA excluding the 3 male subjects was performed. The model was however still significant (one component, CV-ANOVA p = 0.01, R^2^ = 0.4, Q^2^ = 0.2). The OPLS-DA model for clusters 3-5 (patients) showed separation between the clusters based on 26 proteins (VIP>1.0, absolute p(corr) > 0.5). The significant proteins are listed in Supplementary Table 2 in descending order based on VIP value.

**Fig. 3a:**
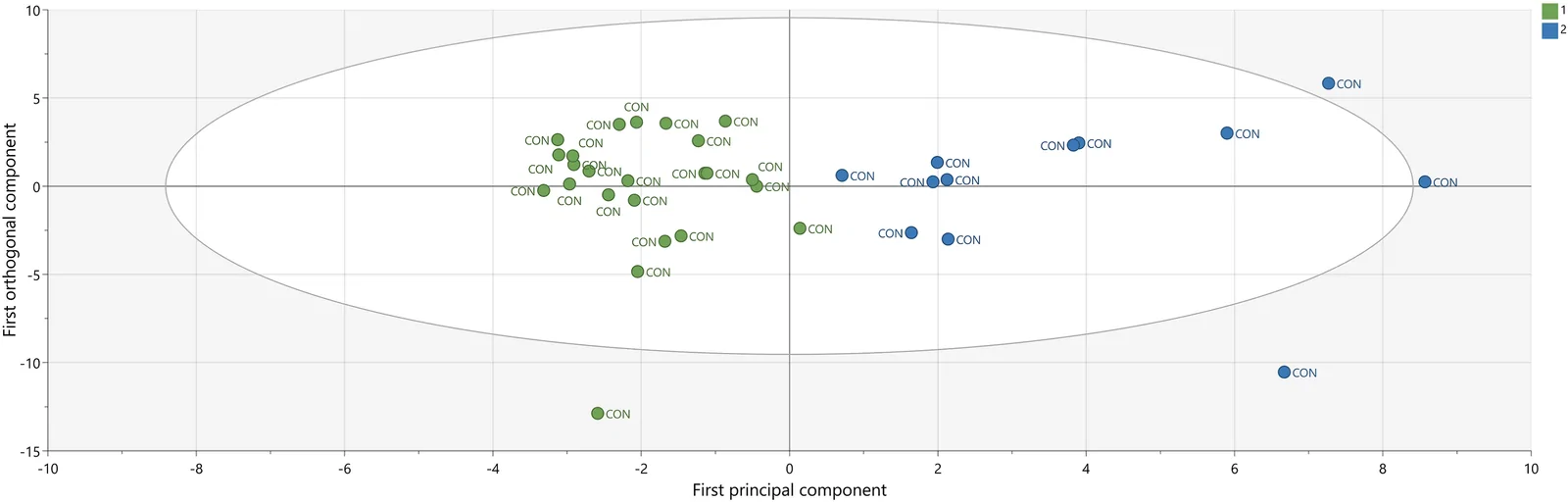
Score plot showing separation between two clusters of healthy subjects using orthogonal partial least square discriminant analysis (OPLS-DA).

**Fig. 3b:**
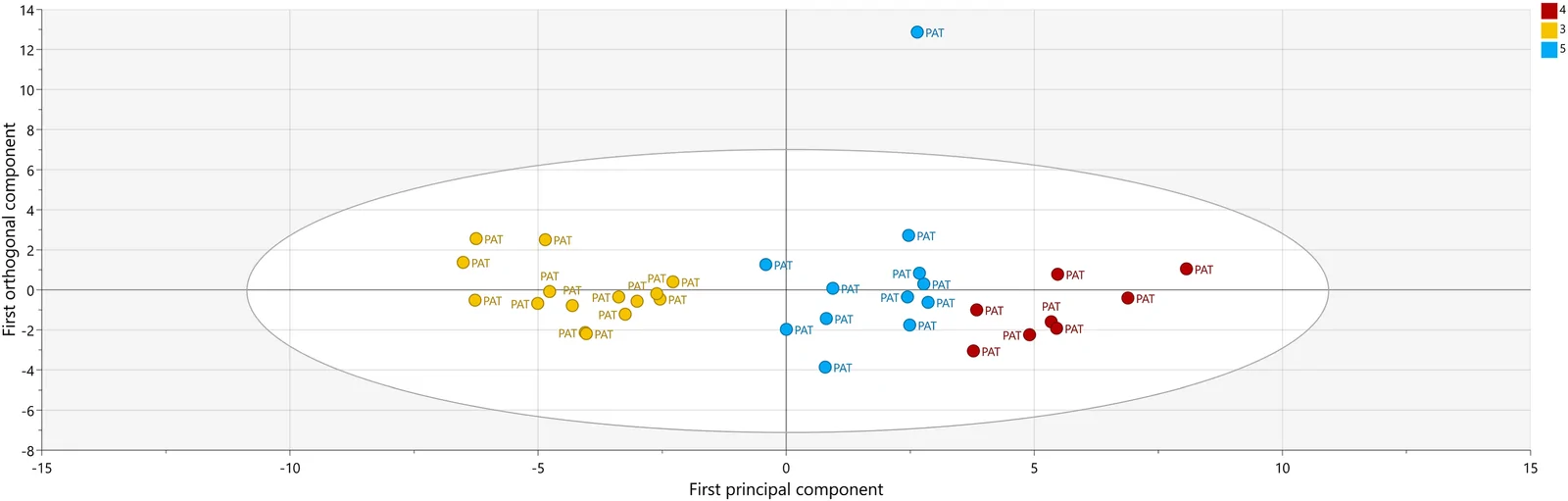
Score plot showing separation between the three clusters of patients using orthogonal partial least square discriminant analysis (OPLS-DA).

### 4.4 Differences in inflammatory proteins between clusters of patients and clusters of healthy subjects

**Fig. 4:**
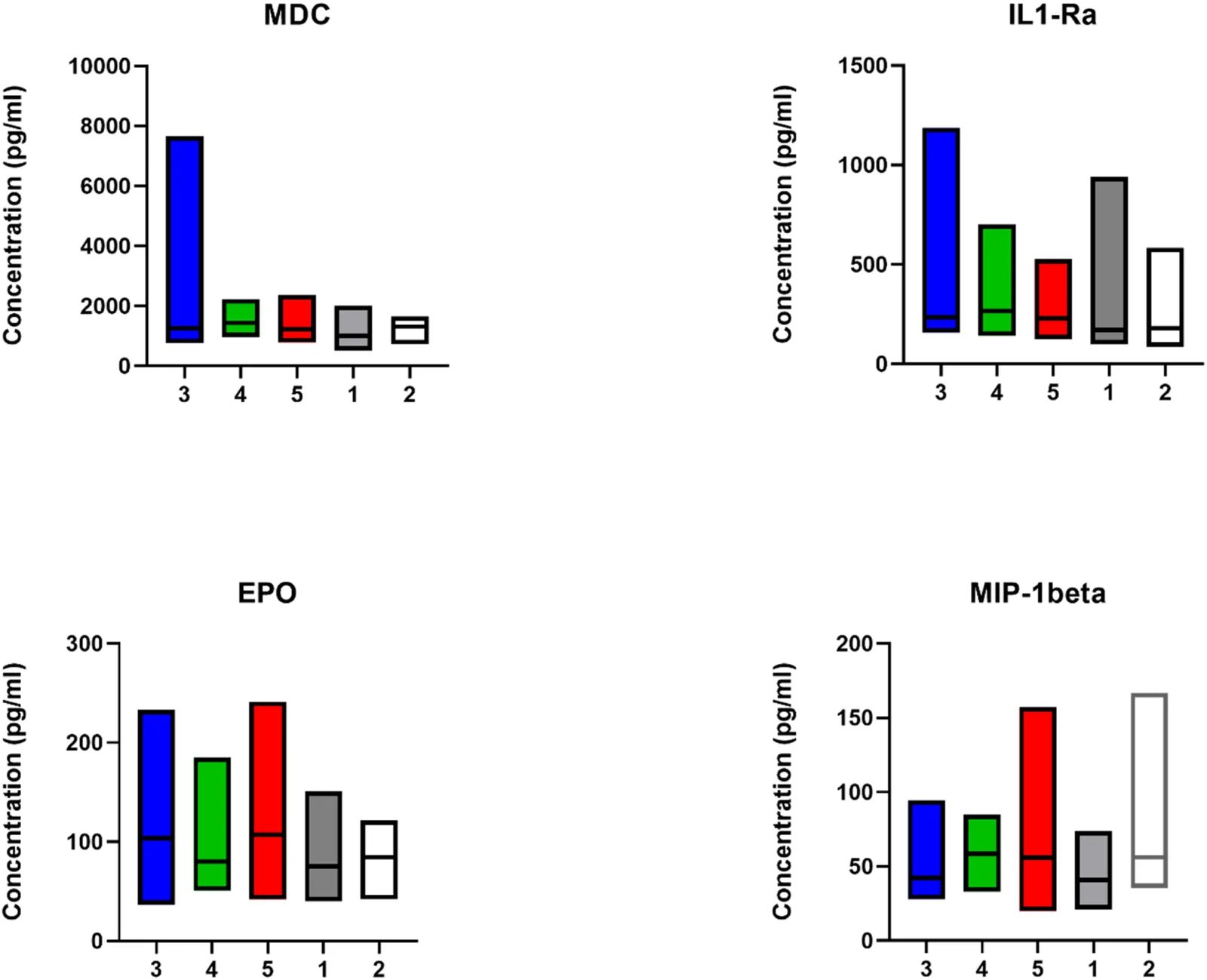
The distribution of the 4 upregulated proteins in the 3 clusters of patients (3, 4, 5) and controls (1 and 2).

OPLS-DA was performed between cluster 1 (healthy controls) and the 3 clusters of patients revealing significant differences between cluster 1 and the 3 clusters of patients. When comparing healthy controls in cluster 2 and the 3 clusters of patients, there was only a significant OPLS-DA model between cluster 2 and 3 (see Supplementary Figs. 1–4 and Supplementary Tables 3–6). Cluster 3 had an overall lower concentration level of inflammatory substances compared to the other clusters. However, there was an exception regarding Erythropoietin (EPO), Interleukin-1 receptor antagonist (IL1RA), Macrophage-Derived Chemokine (MDC) and Macrophage Inflammatory Protein-1beta (MIP-1beta), that were increased in patient group compared to controls in cluster 1 (Supplementary Table 3). The concentrations of these four proteins in the different clusters are shown in Fig. 4.

### 4.5 Pathway analysis of significant proteins in cluster 3

**Fig. 5:**
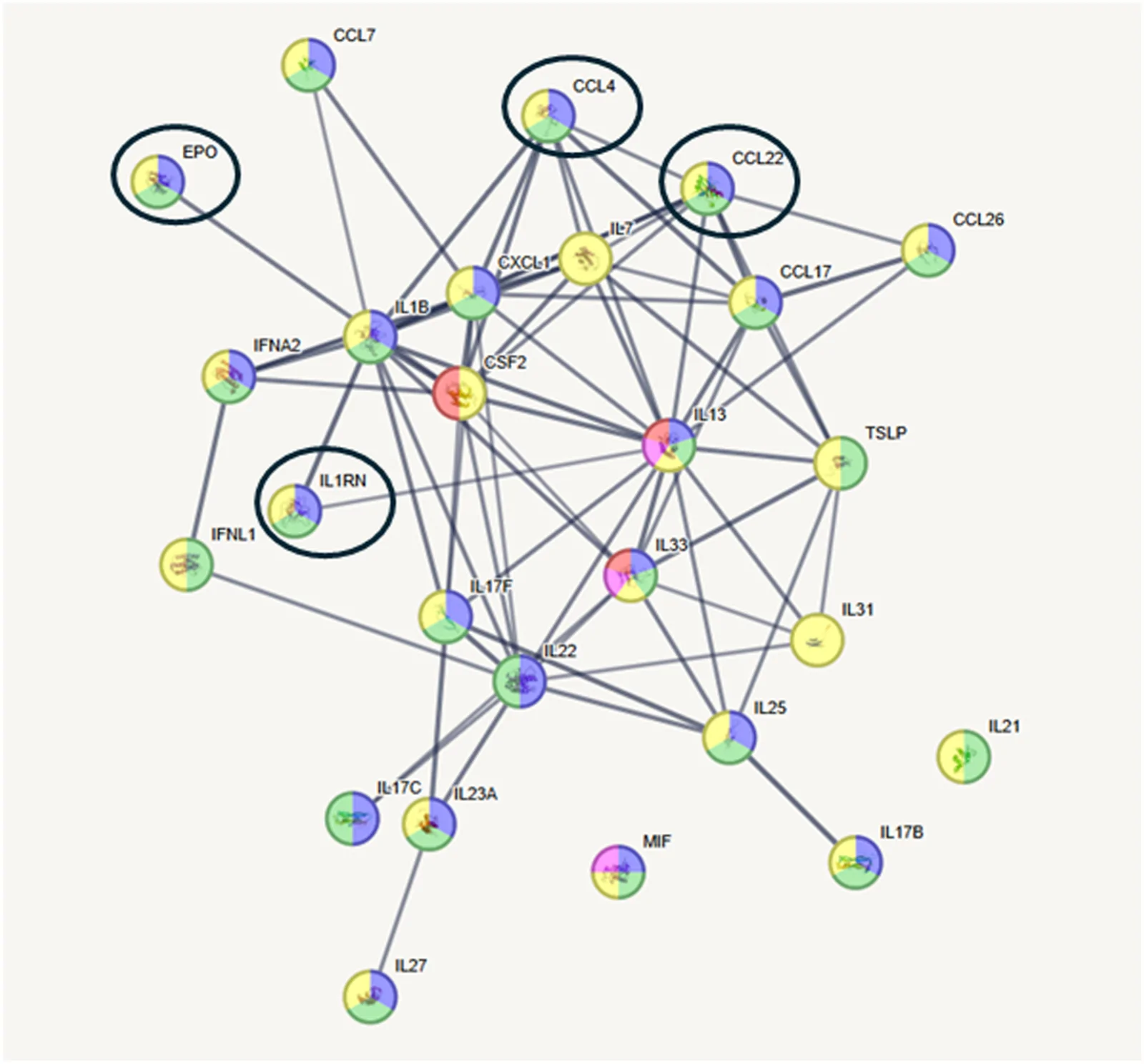
The protein network of the significant proteins that discriminate cluster 3 from healthy subjects. Highlighted are the proteins that are more abundant in cluster 3 compared to cluster 1. Colors in the nodes represent: pink = regulation of macrophage activation, red = macrophage activation, purple = inflammatory response, green = defense response, yellow = immune system process. (For interpretation of the references to colour in this figure legend, the reader is referred to the Web version of this article.)

The biological processes that the significant proteins in cluster 3 (Supplementary Table 3) are involved in were analyzed using protein interaction network analysis that use the corresponding gene according to the UniProt protein data base (https://www.uniprot.org). Fig. 5 shows that the proteins were significantly intercorrelated (PPI enrichment p-value: < 1.0e-16), indicating that the proteins are biologically connected. The corresponding protein names in Fig. 5 are shown in Supplementary Table 7. The identified biological processes were:

1. Regulation of macrophage activation (pink)
2. Macrophage activation (red)
3. Inflammatory response (purple)
4. Defense response (green)
5. Immune system process (yellow)

### 4.6 Biomarker clusters and association with cognitive fatigability and self-reported data

To compare possible effects of inflammatory biomarkers on test of cognitive fatigability and self-reported data in the clusters suggested by the HCA-analysis, a one-way ANOVA was conducted (see Table 4). No significant differences were found regarding cognitive fatigability (*p* = 0.749) between the clusters, indicating that levels of inflammatory biomarkers did not have a significant impact on cognitive fatigability. The results did reveal a significant difference in self-reported general fatigue, as measured by the MFI-20 F (4,66) = 59.19, *p* < 0.001 between the clusters. In addition, there were also differences between the clusters in level of self-rated state fatigue both before F (4, 66) = 16.47, *p* < 0.001 and after F(4, 66) = 18.85), *p* < 0.001 the neuropsychological assessment. The self-reported data also revealed significant differences between the patient clusters regarding number of pain localizations F (2, 29) = 3.46, *p* = 0.045, and perceived helplessness F(2, 29) = 3.42, *p* = 0.046. Significant cluster differences were also found in self-reported data collected from both patients and controls regarding insomnia F (4, 62) = 29.94, *p* < 0.001, depressive symptoms F (4, 63) = 37.73), *p* < 0.001 and anxiety F (4, 63) = 11.51, *p* < 0.001. Level of physical exercise also differed between the clusters F (4, 63) = 4.54,*p* = 0.003, but everyday physical activity and sedentary behavior did not differ significantly between the clusters.

**Table 4:** The mean(SD) of clinical characteristics, and patient reported data.

Self-reported measures	Cluster 1 Mean (SD)	Cluster 2 Mean (SD)	Cluster 3 Mean (SD)	Cluster 4 Mean (SD)	Cluster 5 Mean (SD)	p-value
Cognitive Fatigability	−0.30(6.81)	−1.14(4.41)	−2.38(3.02)	−0.41(3.51)	−1.74(5.51)	0.749
VAS-f 1	25.25(17.79)	21.43(11.29)	65.98(14.36)	44.47(22.73)	55.84(26.09)	**<0.001**
VAS-f2	39.47(25.55)	21.68(14.79)	75.92(13.46)	64.67(13.93)	67.77(16.17)	**<0.001**
Fatigability (VAS1-VAS2)	−14.22(21.32)	−0.255(13.75)	−9.94(14.45)	−20.21(17.27)	−11.93(18.79)	0.140
MFI Total	32.92(9.82)	42.75(10.31)	79.00(6.08)	75.38(8.60)	70.58(14.95)	**<0.001**
MFI General	8.54(3.19)	9.50(2.58)	19.00(1.20)	17.13(1.55)	16.75(2.42)	**<0.001**
MFI Physical	7.38(3.54)	8.25(2.96)	17.27(1.83)	16.63(2.20)	15.25(5.15)	**<0.001**
MFI Mental	8.63(3.23)	10.08(3.03)	15.67(2.38)	15.25(2.66)	15.67(2.96)	**<0.001**
HAD (Anxiety)	3.67(2.76)	6.33(3.63)	11.64(5.75)	9.71(1.38)	6.55(3.11)	**<0.001**
HAD (Depression)	1.25(1.36)	1.17(1.03)	9.50(3.48)	7.43(3.05)	7.00(3.07)	**<0.001**
ISI (Sum initial)	4.33(3.23)	4.17(2.21)	16.86(5.75)	8.86(4.60)	14.80(4.57)	**<0.001**
Physical exercise	4(1.56)	4.42(1.56)	2.50(1.61)	2.29(1.38)	2.64(1.91)	**0.003**
Everyday physical activity	5.54(1.18)	5.58(0.90)	5.07(1.33)	4.29(1.11)	5.0(1.61)	0.144
Sedentary behavior	4.42(1.02)	4.75(0.75)	4.29(1.33)	3.86(1.22)	4.00(1.34)	0.412
Number of pain localizations	n.a	n.a.	22.71(7.41)	14.86(7.93)	15.73(8.33)	**0.045**
Perceived helplessness (PCS)	n.a	n.a	14.36(5.37)	8.43(5.50)	11.45(4.06)	**0.046**

Post-hoc analyses of the significant differences between the clusters showed that cluster 3 reported a higher number of pain localizations than both patient cluster 4 (*p* = 0.039) and patient cluster 5 (*p* = 0.035). Patient cluster 3 also rated higher perceived helplessness than cluster 4 (*p* = 0.016), although non-significant differences compared to patient cluster 5. Insomnia was also rated higher in the patient cluster 3 compared to all other clusters (*p* < 0.001) except patient cluster 5, which was non-significant. Depressive symptoms were significantly different between the patient clusters and controls, but non-significant between the patient clusters. Symptoms of anxiety were reported higher in the patient cluster 3 and differed significantly compared to control cluster 1 (*p* < 0.001), control cluster 2 (*p* = 0.005) and patient cluster 5 (*p* = 0.011), but non-significant compared to patient cluster 4. With regards to self-reported fatigue in the MFI-20 subscales, general fatigue was reported higher in patient cluster 3 compared to control clusters 1 and 2 (both *p* < 0.001) and patient cluster 5 (*p* = 0.023). There were significant differences between patients and controls regarding physical fatigue and mental fatigue (all *p* < 0.001), but no significant differences between the patient clusters emerged. Regarding physical exercise, cluster 3 was significantly less active than cluster 2 (*p* = 0.029). Everyday physical activity and sedentary behavior did not differ between the clusters.

As medications can influence immune function, we also evaluated the use of medications across the different clusters. None of the participants in the control group reported using anti-inflammatory or antidepressant medication. The distribution of SSRI medication was comparable across groups (Cluster 3: 3 individuals; Cluster 4: 2 individuals; Cluster 5: 4 individuals). Regarding anti-inflammatory medication (NSAID), one individual in Cluster 3 and one in Cluster 5 reported use, while two individuals in Cluster 4 reported NSAID use. In addition, one individual in Cluster 5 reported using a TCA. Given the relatively even distribution, the low overall prevalence of medication uses across groups, and small sample size, we did not conduct a sensitivity analysis.

## 5 Discussion

Results of the present study showed no apparent differences in abundance of inflammatory substances between patients and controls as two separate groups. When conducting explorative multivariate PCA analysis of the whole study population with immunological response as the determinant, five specific clusters emerged. Two clusters of controls and three separate clusters of patients. Of the five clusters, patient cluster 3 had an overall lower concentration level of inflammatory substances compared to the other clusters. Patient-reported data also revealed significant differences between patient cluster 3 and the other clusters. Patient cluster 3 reported a higher level of self-rated general fatigue (trait fatigue) as well as self-rated state fatigue, measured both before and after the neuropsychological assessment. In addition, patient cluster 3 also reported higher proportions of additional self-rated symptoms, such as number of pain localizations, perceived helplessness, insomnia, depressive symptoms, and anxiety. This coheres with a recent study reporting inflammatory status within normal range in patients with chronic pain and high levels of self-reported sickness behavior (Åström Reitan et al., 2024). In this study, ratings of sickness behavior contributed more than inflammatory markers in explaining ratings of depression and insomnia. This is contradictory to previously suggested mechanisms proposing a central component in the production of inflammatory biomarkers in the periphery. It is hypothesized that the immune and nervous systems interact reciprocally under many normal and pathological conditions (McMahon et al., 2015). In pain, immune responses (i.e., inflammation) are suggested to modulate neuronal excitability in pain transmission pathways and nerve cells directly, and indirectly, thus modulating immune function (McMahon et al., 2015; Ren et al., 2010). Plausible explanations may include compartmentalized (central) inflammation not reflected in blood, dynamic immune phases (early high inflammation followed by regulatory downregulation), neural sensitization amplifying small immune signals, autonomic and HPA dysregulation, measurement timing/assay limits, and true clinical heterogeneity with multiple phenotypes.

Fatigue is intrinsically problematic to investigate (Kluger et al., 2013) as it often interferes with other conditions in chronic pain, such as depression (Arnold, 2008) and sleep disturbances (Akerstedt et al., 2004), making it difficult to determine whether the reported fatigue is primarily related to pain or secondary to other coexisting conditions (Bartley et al., 2018; Mease et al., 2007; Kurtze et al., 2001). To be able to disentangle possible underlying factors of fatigue, we differentiated between self-rated measurements (general/mental fatigue, state fatigue, self-rated performance fatigue) and objectively measured cognitive fatigability. However, we found no association between levels of inflammatory biomarkers and performance regarding cognitive fatigability level or self-rated state fatigability.

It has been proposed that roughly 50% of people with chronic pain experience clinical levels of sleep disturbance, and approximately 50% of people fulfilling criteria of insomnia experience chronic pain (Todd et al., 2022). Increased levels of inflammatory markers slightly mediate the association between sleep disturbance and depression. Elevated levels of IL-6, IL-1beta and Tumor necrosis factor has been suggested in patients with major depressive disorder vs healthy controls (Gavril et al., 2024). A recent review providing an overview of various biomarkers of importance for the regulation of depression stated that inflammation biomarkers associated with Major Depressive Disorder are IL-2, IL-6, IL-8, IL-10 (TNF) α, interleukin (IL)-1b, and serum FAM19A5 as well as greater levels of the pro-inflammatory cytokine TNF- α (Rimti et al., 2023). Sleep and sleep disturbances are also associated with increased levels of pro-inflammatory biomarkers such as interleukin-1 (IL-1), interleukin-6 (IL-6) and tumor necrosis factor (TNF) (Irwin et al., 2016; Besedovsky et al., 2019). IL-6 is a well described inflammatory substance, maybe due to specific interest for this interleukin, or maybe due to the feasible analytical methodology. Publication bias due to availability of analytical methods and previous publications within the research area may also be contributing factors. Although IL-6 is not differing significantly in its abundance in this specific study, other members of the IL-6 family (Rose-John, 2018) show a difference in abundance. The IL-6 members have the same physiological anti-or pro-inflammatory effect stimulating the inflammatory type 2 cascade. The IL-6 family members identified to differentiate between the clusters in this study were IL 27, IL11, IL31, IL35, IL39, and OncostatinM. Hence, the inflammatory processes activated by IL-6 family members are present.

In our sample, an anticipated higher prevalence of sleep disturbance and depressive symptoms was found in the patient group. There were, however, no associations with levels of inflammatory biomarkers. On the contrary, cluster 3 reported significantly higher levels of sleep disturbance and depressive symptoms but were found to have lower levels of inflammatory biomarkers. Cluster 3 and Cluster 5 rated high levels of insomnia, fulfilling the criteria of clinical insomnia. Interestingly, these two clusters also have higher levels of Erythropoietin (EPO) in common. EPO is considered to increase cognitive performance (Wakhloo et al., 2020; Curto et al., 2024; Ehrenreich et al., 2022; Singh et al., 2023) and serve as a neuroprotective agent, due to its capability to increase restoration and growth of neurons (Zhang et al., 2014) triggering the production of the neuronal growth factor, Brain Derived Neurotrophic Factor (BDNF), and the chemokine Stromal Derived Factor (SDF-1) as well as the enzyme enolase. BDNF has been suggested to be involved in enhanced pain perception (Pezet et al., 2006; Boucher et al., 2001). EPO has in previous studies shown to have a synergistic effect with Neuronal growth factor) (NGF) (Barker et al., 2020) suggested to promote sciatic nerve repair (Zhang et al., 2017). Suggesting a higher abundance of EPO to have a potential role in nerve repair and pain mediation, thus, to be further elucidated in future studies of pain and depression. In addition, injection of EPO has been shown to enhance peripheral nerve recovery by activating macrophage that clear debris and enhance nerve restoration (Said et al., 2021).

This coheres with our results, where the macrophage stimulating chemokines MCP1/CCL2 and MIP-2 also are apparent in a higher concentration in Cluster 3, thus indicative of ongoing repair mechanisms. Also, IL 8, also known as c-x-c motif chemokine ligand 8 (CXCL8) is a chemokine that has been of specific interest in studies of pain (Cunha et al., 1991; Mendieta et al., 2016a). IL-8 is a member of the chemokine family (Russo et al., 2014) with its members (CXCL4) and CXCL2 being identified as more abundant in the present pain clusters, in coherence with previous studies (Gerdle et al., 2017; Bäckryd et al., 2017). Cluster 3 showed lower level of cytokine activation in comparison to cluster 4 and 5. The cytokines of higher abundance in cluster 3 is IL8 and receptors of macrophage activation. Interleukin-8 (IL-8, or CXCL8) primarily acts as a potent pro-inflammatory chemokine, initiating and amplifying inflammatory responses, attracting neutrophils to infection sites. However, it can also exhibit anti-inflammatory effects or be involved in regulating inflammation, depending on context, concentration, and cell type (Qazi et al., 2011). The IL8 secretion is suggested to be induced by other inflammatory cytokines (Matsushima et al., 1989), participates in inflammation and wound healing (Dobreva et al., 2006), is a chemotactic for fibroblasts (Feugate et al., 2002) and also enhances the metabolism of ROS (reactive oxygen species) and increases chemotaxis and the enhanced expression of adhesion molecules (Hechtman et al., 1991). Hence, IL 8 and macrophages have various functions. IL 8 has been found to appear with an increased abundance in studies of low back pain (Dhahri et al., 2025), fibromyalgia (Mendieta et al., 2016b) and major depression (Versel et al., 2024). In studies of the present conditions, the exact function and mechanism of IL8, with its presence, is not clarified. Since, in cluster 3 of the present study, other interleukins such as IL1 and TNF-alpha did not show increased abundance, this pattern might suggest that the roles of IL 8 in these datasets are not primarily proinflammatory. Instead, it may point toward an ongoing anti-inflammatory or tissue repair process, a possibility that warrants further investigation in future research.

The results of the present study suggest that cluster 4 and 5, with a higher proportion of significant inflammatory substances and lower degree of perceived helplessness, insomnia, depressive symptoms and anxiety and discomfort, have a more prominent inflammatory component driving pain mechanisms. Results indicate that cluster 3 may speculatively infer that the pain-response is driven by central pain mechanisms, since the inflammation hypothetically driving the pain mechanisms is not as apparent.

Conclusively, intraindividual variation was identified in the present study by differences in inflammatory responses. Recent publications state that the sensation of pain includes a variety of intraindividual variation (Fillingim, 2017) explained by psychological reactions (Zhu et al., 2023). In the present study the clusters entailed differences in psychological parameters, such as fatigue measurements, insomnia, pain intensity and number of pain locations. The main finding of this study is that to find adequate biomarkers of pain, future studies need to use more refined methodology to be able to separate the nature of the different pain states, as well as adding psychological factors to the methodology.

To date, there is no consensus regarding the specific inflammatory substances involved in the genesis and maintenance of pain, nor with their relation to symptoms related to chronic pain. Although the etiology of chronic pain is still unclear, inflammatory mechanisms have been suggested as contributing factors in the development and maintenance of chronic pain (Ren et al., 2010; Grace et al., 2014). However, as mentioned earlier, there are discrepancies between studies where some highlight elevated cytokine levels in patients with chronic pain, whilst some show no significant differences. Discrepancies in cytokine findings could be due to methodological variations, publication bias, sample variability, or simply representative of the heterogeneity of chronic pain etiology. In addition, one meta-analysis revealed that the majority of cytokines levels in the analyzed studies did not differ between patients and controls (Üçeyler et al., 2011). Our study provides some evidence that patients with chronic pain may not be a homogeneous group and simple patients vs. control studies are perhaps not sufficient to explain underlying inflammatory processes in chronic pain. Thus, underscoring the complexity of conditions involving chronic pain.

### 5.1 Limitations

The present study entails a consecutive selection from a larger population of 200 study subjects. However, although consecutively enrolled, the study population in the present study were subjected to additional exclusion criteria as they were also enrolled in a fMRI sub-study. Those willing to participate in the present study may therefore be less fatigued than those who declined to take part in additional examinations and fMRI investigation. Also, the study population consisted basically exclusively of women and in the younger age span, which limit the generalizability of the results. Interestingly the controls were also not a homogeneous group with regards to abundance of inflammatory biomarkers, whereby the normality in the control group as well as the healthy population may be debatable. We also recognize the lack of data regarding additional variables that may influence the abundance of inflammatory biomarkers (such as smoking, diet and information on menstrual cycle for example). BMI was not registered in the control group, which in hindsight would have been desired. We did not see any significant differences in BMI between the patient clusters, which may indicate that this is not a confounding factor in this sample, i.e. clusters 3-5. Still, for future studies, a well characterized control group would be of importance when analyzing inflammatory biomarkers.

The relatively small sample size in the different clusters is also recognized as a limitation, which may reduce the statistical power to detect significant effects and limit the generalizability of the findings. As a result, the observed associations should be interpreted with caution, and future research with larger cohorts is needed to confirm and extend these results.

With the chosen study design, we have excluded other neurological conditions (including ME/CFS), concussion, and medical sedatives as fatigue-related factors. However, we cannot ensure that the reported fatigue originated specifically from pain. In addition, self-reported fatigue is tightly interlinked with depression and sleep disorders, which is why the three are difficult to separate. For the control group, fatigue was not an exclusion criterion. In an earlier publication (Holmqvist et al., 2024) including the whole study population the general fatigue score in the control group, measured with Multidimensional Fatigue Inventory −20, was 8 (range 4-16) which is equivalent with a normal score in a healthy population (Engberg et al., 2017). However, some of the individuals in the control group scored rather high fatigue levels.

In the present study, medication use was assessed through self-reported data, which may be subject to underreporting due to recall bias or misreporting. Conversely, reliance on medical records does not guarantee actual medication adherence, thereby introducing a potential for overestimation of medication use.

The strength lies in our comparison between chronic pain patients and controls and the association between inflammatory biomarkers, fatigue and other well-known coexisting conditions such as depression and sleep disorders. We also highlight the importance of well-defined studies with comprehensive methodology when studying the relation between pain and inflammation.

## 6 Conclusion

This study identified different clusters of chronic pain patients and controls, visualizing the complexity of the patient population. The results indicate that chronic pain patients constitute a heterogeneous group, and levels of inflammatory biomarkers do not reliably distinguish them from healthy controls. Instead, the findings suggest the presence of distinct subgroups within the chronic pain population, characterized by differing patterns in the association between self-rated fatigue, sleep disturbances, and depression and biomarker levels. There was, however, an association between specific biomarkers indicative of ongoing repair mechanisms and self-rated fatigue as well as several other symptoms. This highlights the complexity regarding the sensation of pain and its connection to inflammatory responses and individual symptom experiences.

## CRediT authorship contribution statement

**Jenny Hadrévi:** Writing – review & editing, Writing – original draft, Visualization, Validation, Methodology, Investigation, Formal analysis, Conceptualization. **Johanna Philipson:** Writing – review & editing, Writing – original draft, Visualization, Project administration, Methodology, Funding acquisition, Formal analysis, Conceptualization. **Britt-Marie Stålnacke:** Writing – review & editing, Validation, Project administration, Methodology, Funding acquisition, Data curation, Conceptualization. **Nils Berginström:** Writing – review & editing, Validation, Project administration, Methodology, Investigation, Funding acquisition, Data curation, Conceptualization. **Monika Löfgren:** Writing – review & editing. **Anna Holmqvist:** Writing – review & editing, Investigation. **Marika C. Möller:** Writing – review & editing, Validation, Project administration, Methodology, Funding acquisition, Data curation, Conceptualization. **Bijar Ghafouri:** Writing – review & editing, Visualization, Validation, Resources, Project administration, Methodology, Funding acquisition, Formal analysis, Data curation, Conceptualization.

## Ethics declaration

Written informed consent to take part in the study and to publish the article has been obtained from all participants or their legal representatives. The privacy rights of participants have been observed.

This study was performed in compliance with relevant laws, regulatory frameworks and guidelines where the research took place. This study was approved by the Swedish Ethics Review Board. (Approval No. Dnr 2018/424-31; 2018/1235–32; 2018/2395–32; 2019– 66148; 2022-02838- 02.)

## Funding

This study was funded by Karolinska Institutet, Department of Clinical Sciences, the Promobilia Foundation (no. A22056), by the research and development fund granted by Vasterbotten County Council, and through a regional agreement between Umeå University and Vasterbotten County Council (ALF).

## Declaration of competing interest

The authors declare the following financial interests/personal relationships which may be considered as potential competing interests:

Johanna Philipson reports financial support was provided by Region Västerbotten. Marika Moller reports financial support was provided by Karolinska Institutet Department of Clinical Neuroscience. Britt-Marie Stalnacke reports was provided by Karolinska Institutet Department of Neuroscience. Anna Holmqvist reports financial support was provided by Promobilia foundation. Nils Berginstrom reports financial support was provided by Region Västerbotten. If there are other authors, they declare that they have no known competing financial interests or personal relationships that could have appeared to influence the work reported in this paper.

## Data Availability

Data will be made available on request.
