# Supplementary material for: An explorative cross-sectional study on the association between immunological biomarkers in patients with chronic pain
Source: Brain Behav Immun Health. 2026 Aug 29;56:101339. doi: 10.1016/j.bbih.2026.101339 (PMC13553578; doi:10.1016/j.bbih.2026.101339)
Supplement: Supplementary file 1 [file mmc1.docx]

# Supplementary material

**Supplementary figure 1.** OPLS-DA model showing significant separation between cluster 1 and 5 (two components, CV-ANOVA p=0.01, R2= 0.7, Q2= 0.4).

**Supplementary figure 2.** OPLS-DA model showing significant separation between cluster 1 and 4 (one component, CV-ANOVA p=0.002, R2= 0.5, Q2= 0.3).

**Supplementary figure 3.** OPLS-DA model showing significant separation between cluster 1 and 3 (two components, CV-ANOVA p=0.0005, R2= 0.8, Q2= 0.5).

**Supplementary figure 4.** OPLS-DA model showing significant separation between cluster 2 and 3 (two components, CV-ANOVA p=0.002, R2= 0.9, Q2= 0.5).

**Supplementary Table 1**. Significant proteins that contribute to separation between cluster 1 (n=24) and cluster 2 (n=12). All proteins were significantly higher in cluster 2 compared to cluster 1.

| **PROTEINS** | **VIPPRED** | **P(CORR)** |
| --- | --- | --- |
| **IL-22** | 1.930 | -0.866 |
| **IL-21** | 1.853 | -0.832 |
| **IL-23** | 1.821 | -0.817 |
| **IL-17E** | 1.803 | -0.811 |
| **IL-29** | 1.798 | -0.807 |
| **IL-17F** | 1.794 | -0.805 |
| **TSLP** | 1.773 | -0.796 |
| **IL-7** | 1.719 | -0.771 |
| **MCP-4** | 1.719 | -0.771 |
| **IL-33** | 1.688 | -0.759 |
| **IL-27** | 1.657 | -0.745 |
| **IL-3** | 1.654 | -0.740 |
| **IL-13** | 1.588 | -0.711 |
| **GRO-Α** | 1.535 | -0.689 |
| **TARC** | 1.423 | -0.642 |
| **MCP-3** | 1.392 | -0.621 |
| **ENA-78** | 1.305 | -0.583 |
| **IL-17C** | 1.305 | -0.584 |
| **IL-8** | 1.255 | -0.562 |
| **TPO** | 1.139 | -0.514 |
| **MIP-3Α** | 1.130 | -0.502 |
| **IL-17B** | 1.095 | -0.487 |
| **IL-31** | 1.080 | -0.487 |
| **EOTAXIN** | 1.062 | -0.470 |
| **VEGF-A** | 1.029 | -0.460 |

**Supplementary Table 2.** significant proteins that contribute to separation between the 3 clusters of patients. Mean values in picogram/ml.

| PROTEINS | VIP_PRED_ | P(CORR) | Cluster 3  mean(SD) | Cluster 4  mean (SD) | Cluster 5  mean (SD) |
| --- | --- | --- | --- | --- | --- |
| IL-29 | 1.91 | -0.94 | 39.22 (13.27) | 111.76 (15.11) | 74.61 (17.00) |
| IL-23 | 1.90 | -0.94 | 63.37 (28.14) | 201.48 (31.54) | 138.86 (37.52) |
| IL-21 | 1.89 | -0.94 | 158.46 (56.34) | 451.02 (64.06) | 307.64 (77.35) |
| IL-17E | 1.88 | -0.93 | 29.52 (10.59) | 83.82 (15.62) | 56.65 (14.46) |
| IL-31 | 1.87 | -0.92 | 170.68 (39.23) | 309.56 (24.91) | 246.81 (32.04) |
| IL-22 | 1.85 | -0.91 | 7.50 (2.67) | 21.41 (3.71) | 13.73 (3.63) |
| IL-17F | 1.83 | -0.91 | 3654.98 (1057.37) | 9645.62 (1927.32) | 6429.44 (1423.23) |
| TSLP | 1.82 | -0.90 | 21.34 (8.35) | 55.51 (11.16) | 39.13 (10.26) |
| IL-33 | 1.82 | -0.90 | 68.91 (23.23) | 193.96 (21.79) | 131.52 (42.92) |
| IL-27 | 1.76 | -0.87 | 509.55 (148.83) | 1118.50 (163.30) | 754.01 (161.78) |
| MCP-3 | 1.67 | -0.82 | 3.41 (0.97) | 6.51 (1.39) | 5.87 (1.41) |
| IL-17C | 1.59 | -0.79 | 3.04 (1.17) | 7.25 (2.27) | 6.20 (1.81) |
| MCP-4 | 1.54 | -0.76 | 41.46 (7.05) | 90.40 (19.50) | 71.61 (29.33) |
| IL-17D | 1.51 | -0.74 | 10.81 (3.63) | 24.57 (7.57) | 20.21 (7.72) |
| IL-8 | 1.46 | -0.72 | 2.34 (0.61) | 4.63 (1.58) | 3.65 (0.69) |
| IL-13 | 1.44 | -0.71 | 3.24 (1.47) | 6.03 (1.21) | 5.849 (1.76598) |
| MIF | 1.34 | -0.66 | 44197.87 (8860.27) | 69132.33 (17660.30) | 55235.20 (11061.22) |
| GRO-Α | 1.33 | -0.66 | 87.39 (28.48) | 209.86 (93.93) | 241.89 (98.38) |
| IL-3 | 1.29 | -0.64 | 11.16 (3.57) | 21.26 (6.23) | 20.53 (9.39) |
| IL-7 | 1.25 | -0.62 | 2.07 (0.71) | 3.70 (0.82) | 4.62 (1.79) |
| I-TAC | 1.11 | -0.56 | 22.31 (7.26) | 38.07 (12.64) | 36.18 (13.39) |
| TARC | 1.08 | -0.53 | 33.25 (13.69) | 86.16 (47.00) | 79.66 (59.58) |
| TPO | 1.03 | -0.51 | 346.18 (53.29) | 433.76 (87.21) | 391.06 (79.49) |
| MCP-1 | 1.01 | -0.49 | 130.80 (22.67) | 200.19 (43.50) | 156.80 (62.21) |
| EOTAXIN | 1.01 | -0.50 | 162.14 (29.39) | 213.67 (56.93) | 235.39 (52.90) |
| ENA-78 | 1.00 | -0.50 | 156.22 (74.64) | 334.31 (208.46) | 430.82 (237.83) |

**Supplementary table 3**. Significant proteins that differ between cluster 1 and cluster 3. The minus sign of p(corr) indicates that concentration of the protein was downregulated in cluster 3 compared to cluster 1.

| Proteins | VIPpred | p(corr) |
| --- | --- | --- |
| IL-31 | 2.049 | -0.726 |
| IL-13 | 1.700 | -0.602 |
| MCP-3 | 1.675 | -0.593 |
| IL-33 | 1.653 | -0.586 |
| IL-29 | 1.626 | -0.576 |
| IL-17E | 1.626 | -0.576 |
| IL-23 | 1.582 | -0.561 |
| IL-21 | 1.577 | -0.559 |
| IL-17F | 1.576 | -0.559 |
| IL-17C | 1.499 | -0.532 |
| TSLP | 1.447 | -0.512 |
| IL-22 | 1.429 | -0.507 |
| MDC | 1.419 | 0.503 |
| IL-17A/F | 1.391 | -0.562 |
| IL-27 | 1.363 | -0.483 |
| IL-17B | 1.351 | -0.478 |
| GM-CSF | 1.343 | -0.501 |
| IL-1RA | 1.326 | 0.471 |
| MIF | 1.318 | -0.466 |
| IFN-α2a | 1.293 | -0.484 |
| EPO | 1.287 | 0.456 |
| IL-7 | 1.277 | -0.453 |
| GRO-α | 1.236 | -0.439 |
| Eotaxin-3 | 1.234 | -0.439 |
| MIP-1β | 1.221 | 0.433 |
| IL-1β | 1.186 | -0.453 |
| TARC | 1.155 | -0.411 |

**Supplementary table 4**. Significant proteins that differ between cluster 1 and cluster 4. All proteins were upregulated in cluster 4 compared to cluster 1, except IL-1β.

| Proteins | VIP | p(corr) |
| --- | --- | --- |
| IL-29 | 1.812 | 0.932 |
| IL-33 | 1.799 | 0.925 |
| IL-21 | 1.797 | 0.924 |
| IL-23 | 1.790 | 0.920 |
| IL-17E | 1.763 | 0.907 |
| IL-22 | 1.763 | 0.907 |
| TSLP | 1.735 | 0.892 |
| IL-27 | 1.728 | 0.889 |
| IL-17F | 1.716 | 0.883 |
| MCP-4 | 1.637 | 0.842 |
| IL-3 | 1.595 | 0.820 |
| IL-17C | 1.579 | 0.812 |
| MCP-1 | 1.403 | 0.721 |
| MCP-3 | 1.390 | 0.715 |
| IL-17B | 1.342 | 0.690 |
| IL-8 | 1.289 | 0.663 |
| TARC | 1.283 | 0.660 |
| MCP-2 | 1.237 | 0.635 |
| IL-13 | 1.236 | 0.635 |
| IL-17D | 1.219 | 0.626 |
| IL-7 | 1.200 | 0.617 |
| MIP-1β | 1.194 | 0.613 |
| I-TAC | 1.116 | 0.574 |
| MIP-3α | 1.114 | 0.573 |
| IL-1β | 1.112 | -0.626 |
| MDC | 1.102 | 0.567 |
| GRO-α | 1.101 | 0.566 |
| TPO | 1.081 | 0.556 |
| IL-1RA | 1.074 | 0.552 |
| ENA-78 | 1.033 | 0.531 |
| IL-31 | 1.018 | 0.524 |

**Supplementary table 5**. Significant proteins that differ between cluster 1 and cluster 5. All proteins were upregulated in cluster 5 compared to cluster 1.

| Primary ID | VIPpred | p(corr) |
| --- | --- | --- |
| TNF-α | 2.101 | 0.731 |
| I-TAC | 1.913 | 0.666 |
| ENA-78 | 1.904 | 0.662 |
| VEGF-A | 1.796 | 0.627 |
| MIP-3α | 1.748 | 0.610 |
| IL-7 | 1.744 | 0.607 |
| Fractalkine | 1.705 | 0.592 |
| GRO-α | 1.695 | 0.589 |
| SDF-1α | 1.648 | 0.574 |
| Il-17A | 1.510 | 0.526 |
| CTACK | 1.387 | 0.482 |
| EPO | 1.279 | 0.447 |
| TRAIL | 1.257 | 0.438 |
| IL-6 | 1.247 | 0.432 |
| MCP-2 | 1.234 | 0.430 |
| IFN-β | 1.217 | 0.416 |
| IL-17A/F | 1.215 | 0.469 |
| MCP-4 | 1.193 | 0.414 |
| IL-3 | 1.190 | 0.415 |
| IL-8 | 1.190 | 0.416 |

**Supplementary table 6**. Significant proteins that differ between cluster 2 and cluster 3. The minus sign of p(corr) indicates that concentration of the protein was downregulated in cluster 3 compared to cluster 2.

| Proteins | VIPpred | p(corr) |
| --- | --- | --- |
| IL-31 | 1.674 | -0.882 |
| IL-21 | 1.671 | -0.880 |
| IL-17E | 1.670 | -0.880 |
| GRO-α | 1.648 | -0.870 |
| IL-22 | 1.648 | -0.869 |
| IL-29 | 1.646 | -0.867 |
| IL-17F | 1.641 | -0.864 |
| IL-23 | 1.630 | -0.859 |
| IL-7 | 1.626 | -0.857 |
| MCP-3 | 1.603 | -0.843 |
| IL-13 | 1.597 | -0.841 |
| TSLP | 1.573 | -0.828 |
| IL-33 | 1.559 | -0.822 |
| IL-8 | 1.499 | -0.791 |
| MCP-4 | 1.499 | -0.789 |
| IL-27 | 1.464 | -0.771 |
| TARC | 1.404 | -0.745 |
| IL-3 | 1.268 | -0.667 |
| ENA-78 | 1.240 | -0.650 |
| IL-17C | 1.232 | -0.649 |
| IL-17D | 1.161 | -0.610 |
| IL-17B | 1.135 | -0.596 |
| IFN-α2a | 1.107 | -0.609 |
| MIF | 1.055 | -0.554 |
| VEGF-A | 1.040 | -0.548 |
| Eotaxin-3 | 1.013 | -0.533 |

**Supplementary Table 7**. Clarification of the name of gen names in the pathway analysis.

| Protein name | Gen name |
| --- | --- |
| IL-31 | IL31 |
| IL-13 | IL13 |
| MCP-3 | CCL7 |
| IL-33 | IL33 |
| IL-29 | IFNL1 |
| IL-17E | IL25 |
| IL-23 | IL23A |
| IL-21 | IL21 |
| IL-17F | IL17F |
| IL-17C | IL17C |
| TSLP | TSLP |
| IL-22 | IL22 |
| MDC | CCL22 |
| IL-17A/F | IL17F |
| IL-27 | IL27 |
| IL-17B | IL17B |
| GM-CSF | CSF2 |
| IL-1RA | IL1RN |
| MIF | MIF |
| IFN-α2a | IFNA2 |
| EPO | EPO |
| IL-7 | IL7 |
| GRO-α | CXCL1 |
| Eotaxin-3 | CCL26 |
| MIP-1β | CCL4 |
| IL-1β | IL1B |
| TARC | CCL17 |
